# Breast conservation surgery versus total mastectomy among women with localized breast cancer in Soweto, South Africa

**DOI:** 10.1371/journal.pone.0182125

**Published:** 2017-08-10

**Authors:** Herbert Cubasch, Maureen Joffe, Paul Ruff, Donald Dietz, Evan Rosenbaum, Nivashni Murugan, Ming Tsai Chih, Oluwatosin Ayeni, Caroline Dickens, Katherine Crew, Judith S. Jacobson, Alfred Neugut

**Affiliations:** 1 Department of Surgery, Faculty of Health Sciences, University of the Witwatersrand, Johannesburg, South Africa; 2 Batho Pele Breast Unit, Chris Hani Baragwanath Academic Hospital, Johannesburg, South Africa; 3 Wits Health Consortium, Johannesburg, South Africa; 4 Department of Paediatrics, Faculty of Health Sciences, University of the Witwatersrand, Johannesburg, South Africa; 5 Division of Medical Oncology, Department of Internal Medicine, Faculty of Health Sciences, University of the Witwatersrand, Johannesburg, South Africa; 6 Department of Internal Medicine, Faculty of Health Sciences, University of the Witwatersrand, Johannesburg, South Africa; 7 Department of Medicine, College of Physicians and Surgeons, Columbia University, New York, New York, United States of America; 8 Herbert Irving Comprehensive Cancer Center, College of Physicians and Surgeons, Columbia University, New York, New York, United States of America; 9 Department of Epidemiology, Mailman School of Public Health, Columbia University, New York, New York, United States of America; Tata Memorial Centre, INDIA

## Abstract

**Purpose:**

Breast conserving surgery (BCS) has become the preferred surgical option for the management of patients with nonmetastatic breast cancer in high-income countries. However, little is known about the distribution and determinants of BCS in low-and middle-income countries, especially those with high HIV prevalence.

**Methods:**

We compared demographic and clinical characteristics of female patients who received BCS and those who received total mastectomy (TM) for nonmetastatic invasive carcinoma of the breast in Soweto, South Africa, 2009–2011. We also developed a multivariable logistic regression model of predictors of type of surgery.

**Results:**

Of 445 patients, 354 (80%) underwent TM and 91 (20%) BCS. Of 373 patients screened for HIV, 59 (15.8%) tested positive. Eighty-two of 294 patients with stage I/II disease (28%), but just 9 of 151 (6%) with stage III disease had BCS (p<0.001). All women who received BCS (except for seven who received completion mastectomy within 6 weeks of BCS) and 235 (66.4%) women who received TM were referred for radiation therapy (RT). In our multivariable analysis, age group 50–59 years (OR = 2.28, 95% CI = 1.1–4.8) and ≥70 years (OR = 9.55, 95% CI = 2.9–31.2) vs. age group <40 years, stage at diagnosis (stage II (OR = 3.79, 95% CI = 1.6–8.2) and stage III (OR = 27.8, 95% CI = 9.0–78.8) vs. stage 1, HIV (HIV positive (OR = 3.19, 95% CI = 1.3–7.9) vs. HIV negative) and HER2-enriched subtype (OR = 3.50, 95% CI = 1.2–10.1) vs. triple negative were independently associated with TM.

**Conclusion:**

TM was more common than BCS among patients with nonmetastatic breast cancer in Soweto, not only among patients with locally advanced disease at diagnosis, but also among women with stage I and II disease.

## Introduction

Breast cancer is the most common cancer affecting women. According to GLOBOCAN, 1.6 million women are diagnosed with the disease worldwide, each year, and 521,000 die with it[1]. High-income countries (HICs)have higher incidence rates of breast cancer than low- and middle-income countries (LMICs), such as those in sub-Saharan Africa, but better survival. Sub-populations within countries also differ in incidence and mortality[2, 3]. The poor outcomes observed in low-income populations may reflect advanced stage at presentation, as well as inconsistent adherence and lack of access to state-of-the-art treatment. In addition, comorbidities, such as HIV/AIDS, may make disease progression more rapid and treatment more complex.

The overall prevalence of HIV infection in South Africa is estimated at 19.2%[4]. A national program to provide anti-retroviral therapy (ART) for the treatment of HIV/AIDS was launched in 2004, leading to significant improvement in life expectancy[5]. Meanwhile, the incidence of cancers common in HICs, including breast cancer, has risen. South Africa’s pathology based National Cancer Registry reports that breast cancer incidence rose from 5280 cases in 2000, accounting for 18.8% of cancers in women, to 7086 cases in 2011, accounting for 21.5% of cancers [6, 7].

Landmark studies by Veronesi *et al* (1981)[8] and by Fisher *et al*. (1985) [9] demonstrated that patients who received breast conserving surgery (BCS) and radiation therapy (RT) did not differ in overall survival from those who received total mastectomy (TM) for the treatment of localized invasive breast cancer (although they had higher rates of local recurrence).[9] Thereafter, BCS followed by RT came into routine use and became a preferred option. Data accrued between 1985 and 2002 continued to show equivalent overall survival rates for patients undergoing TM and those receiving BCS plus RT.[10, 11]. Meanwhile, TM came to be combined with primary or secondary breast reconstruction options; innovative oncoplastic breast reconstructive procedures[12] were found to improve cosmetic outcomes over those of the original quadrantectomy or lumpectomy procedures while maintaining oncological safety.

A review of Surveillance, Epidemiology, and End Results (SEER 2010) data from the United States showed that 95.4% of patients undergoing BCS also received adjuvant RT; RT receipt was associated with surgery type, age, comorbidities, income, patient desire to avoid RT, and level of surgeon involvement in the decision.[13] It was also associated with insurance coverage.[14]

Patients with breast cancer present with more advanced disease in South Africa than in the United States. Reasons may be poorer patient awareness, lack of population screening programs, cultural barriers, and poor access to a sometimes dysfunctional health care system[15]. Fears of mutilation and poor treatment outcomes may also delay diagnosis. Although introducing BCS as an option and discussing its pros and cons with patients is challenging and adds complexity to an already stressed health system, we believe it is necessary to address our patients’ fears and expectations.

South Africa has a high prevalence of HIV, but most patients with HIV have access to ART[4]. The choice of surgical procedures at our institution, Chris Hani Baragwanath Academic Hospital (CHBAH) in Soweto, South Africa, is usually not influenced by the HIV status of our patients, although we prefer TM for weak and cachectic patients who are candidates for surgery.

We previously analyzed HIV status and breast cancer presentation among patients diagnosed at CHBAH. Our results indicated that the prevalence of HIV among breast cancer patients was similar to that among women in the general population, and that breast tumor characteristics were not associated with HIV status.[16] We now have investigated variations in surgical practice and their possible associations with clinical and demographic factors (including HIV status) among breast cancer patients at CHBAH. In addition, we assessed early reoperations in the context of recently changed international margin guidelines.

## Methods

### Setting and patients

CHBAH is the largest tertiary care hospital in South Africa. It is located in Soweto, south-west of Johannesburg, and primarily serves the 3 million black and low-income residents of Soweto and its surrounding neighborhoods. Patients who present with a breast lesion at CHBAH undergo triple assessment (clinical examination, imaging and histopathology), and those diagnosed with breast cancer receive their surgery and follow-up care at CHBAH. However, for chemo- and radiation therapy, patients must travel to Charlotte Maxeke Academic Hospital (CMJAH), 18 kilometers from CHBAH.

At CHBAH, the physical retrieval of patient records is often problematic and time-consuming for clinicians. Therefore, in 2006 the Surgical Breast Unit established its own electronic database of patient information in. In 2008, the unit was registered with the International Breast Centres Network and developed a standardized approach to breast cancer diagnosis, treatment and follow up, coordinated via a weekly multidisciplinary oncology meeting attended by cancer surgeons, medical and radiation oncologists, and palliative care practitioners.

For this study, we reviewed the records of all the female patients in the database who were newly diagnosed with invasive carcinoma of the breast from January 1, 2009 through December 31, 2011.[17] The study was approved by the Wits Human Research Ethics Committee (Medical).

We excluded from our analysis 46 patients who had stage IV (metastatic) disease at diagnosis and 110 patients who did not undergo surgery for other reasons (patients with nonresectable cancers, including those who could not be down-staged with systemic treatment and those who defaulted or died during primary systemic treatment).

### Surgical procedures

Surgery remains the main treatment modality for localized breast cancer. Our approach was to consider the feasibility for BCS in discussion with each patient. We found the indication to be a delicate balance between technical feasibility and safety, individual patient preferences, and patients’ psychosocial and socioeconomic circumstances. Where possible, we addressed the barrier of additional out-of-pocket patient transport costs associated with mandatory RT at CMJAH by helping patients apply for temporary disability grants. We performed few primary and delayed post-mastectomy reconstructions due to resource constraints (shortage of operating theater time, inconsistent supply of breast implants, and limited availability of plastic surgery services) and consequently limited patient demand. We almost always performed level 1 and level 2 oncoplastic procedures (rather than simple lumpectomies)[18]. We were therefore able to treat relatively large lesions with predictably acceptable cosmetic outcomes, while avoiding the morbidity associated with distant flaps.

During the period when our study patients were diagnosed and treated, it was not always easy to deliver surgical services. CHBAH frequently faced logistic challenges, shortages of linen, electricity, water and consumable supplies (e.g., drains, sutures, medicines). In 2009, mammography services were locally unavailable for a month due to machine breakdown, and in 2010, some of our health workers participated in a prolonged public sector strike that brought service delivery to a near standstill and subjected non-striking staff to threats of violence. Despite these problems, most patients who required surgery were operated on within two weeks of a confirmed diagnosis.

Many of our patients were morbidly obese, and many had very large breasts [19]. When such patients required TM, they were routinely offered contralateral reduction mastopexy. Oncoplastic procedures, particularly bilateral breast reductions, were appreciated by patients, facilitated homogeneity, and enhanced the effectiveness of RT[20].

In the choice of procedure, we considered the quadrant of the lesion and the size of the breast, and we limited ourselves to a few easily reproducible and standardized oncoplastic procedures. For inner and outer upper quadrant lesions, we used segmentectomies with parenchymal flaps [12]. Lower inner and outer quadrant lesions were preferably addressed with modified Wise reduction patterns[21]. For large-breasted patients, the preferred option was oncoplastic bilateral reduction using the Wise pattern. Because we used a two-team approach (one team operating on the right breast and the other on the left, simultaneously), these procedures took no more time than routine mastectomies. This approach also enabled senior surgeons to supervise junior staff closely, to reinforce training and team building.

For patients who had very large lesions that did not allow for primary skin closure (usually following primary chemotherapy), we preferred to use thoraco-epigastric abdominal wall advancement flaps, which lead to less morbidity than latissimus dorsi flaps as well as taking less operating time.

We referred patients with locally advanced disease for primary systemic treatment and re-evaluated them post-treatment for potential resectability.

During the study period, we performed sentinel lymph node dissection on patients with a T1 or small T2 lesion and a clinical N0 axilla. In this series, due to exclusion of patients with primary lesions greater than 4cm, irregular availability of radioactive tracers or nuclear medicine services, or lymphadenopathy associated with HIV, fewer than 10% of patients had a sentinel node biopsy. When the sentinel node was found to be positive or if the axilla intra-operatively was highly suspicious, a level 1 and partial level 2 axillary node dissection was performed. Intraoperative frozen sections were rarely available. Because of the high prevalence of HIV infection in this patient population, patients were often found to have histologically cancer-negative reactive lymph nodes.

The average post-operative hospital stay was two days, and patients were discharged with a closed suction drain and followed up weekly in a specialized dressing clinic.

### Data and statistical analysis

Data were extracted from the patients’ electronic medical records. The variables recorded were patient age, race, menopausal status, year of diagnosis, Manchester staging, diagnosis on histopathology, HIV status, histopathology grade, receptor (estrogen, progesterone, HER2) status, tumor size, type of first surgery, recurrence status, time to recurrence, and vital status; Ki-67 analyses were not available during this period. In addition, the records included only limited demographic data and no data on socioeconomic status.

We compared patients who received BCS to those who received TM for early and locally advanced breast cancers. We used chi-square tests to evaluate the statistical significance of differences in frequency distributions of the categorical variables. We then developed multivariable logistic regression models of factors associated with type of surgery, based on the results of the univariate analyses. Our initial hypothesis was that HIV status would be associated with type of surgery. We included in the model factors that we thought might be associated with HIV status and type of surgery (including but not limited to those with p-values <0.1) and were not in the causal pathway, consistent with the standard definition of confounding.

## Results

Of 445 women included in the study, 354 (79.6%) underwent TM and 91 (20.4%) BCS. Of 330 patients who received surgery as the first (initial) treatment modality, 246 (74.5%) received TM and 84 (25.5%) BCS. Of 115 who received surgery following systemic treatment, 108 (93.9%) had TM and 7 (6.1%) BCS (p<0.001) (Table 1).

**Table 1:** Demographic and clinical characteristics of patients who underwent surgery at CHBAH, Soweto, South Africa, 2009–2011, by type of first surgery.

	TM		BCS		Total	
	N	%	N	%	N	%
Totals^a^	354	79.6	91	20.4	445	100.0
Age group						
18–39	55	15.5	20	22.0	75	16.9
40–49	79	22.3	22	24.2	101	22.7
50–59	100	28.2	31	34.1	131	29.4
60–69	61	17.3	13	14.2	74	16.6
70 and above	59	16.7	5	5.5	64	14.4
Race/ethnicity						
Black	325	91.8	82	90.1	407	91.9
Asian	6	1.7	4	4.4	10	2.1
Mixed/Coloured	13	3.7	1	1.1	14	3.0
White	10	2.8	4	4.4	14	3.0
Stage at diagnosis						
I	17	4.8	15	16.5	32	7.2
II	195	55.1	67	73.6	262	58.9
III	142	40.1	9	9.9	151	33.9
Tumor grade*						
1	38	10.9	11	12.1	49	11.2
2	162	45.7	41	45.1	203	45.6
3	139	39.1	37	40.7	176	39.4
Histology**						
Invasive ductal	330	93.2	88	96.7	418	93.9
Invasive lobular	16	4.5	2	2.2	18	4.0
Mixed ductal and lobular	2	0.6	1	1.1	3	0.7
Others	3	0.8	0	0.0	3	0.7
Intrinsic subtypes***						
Luminal A	27	8.2	10	10.9	39	8.8
Luminal B	207	57.9	51	56.0	256	57.5
HER2 enriched	48	13.6	6	6.6	54	12.1
Triple negative	61	17.2	21	23.1	82	18.4
ER and PR expression†						
ER positive alone	44	12.4	10	11.0	54	12.1
ER and PR positive	179	50.6	46	50.5	225	50.6
PR positive alone	14	4.0	6	6.6	20	4.5
ER and PR negative	116	32.8	29	31.9	145	32.6
HIV status on blood test 6 weeks after diagnosis‡						
Negative	245	69.2	69	75.8	314	70.6
Positive	49	13.8	10	11.0	59	13.3
Lymphovascular invasion↨					0	
Present	145	41.0	25	27.5	170	38.2
Absent	151	42.7	48	52.7	199	44.7
Closest invasive marginsΔ						
<1mm	30	8.5	13	14.3	44	9.9
≥1mm & <5mm	46	13.0	17	18.7	64	14.4
5mm—10mm	66	18.6	25	27.5	91	20.4
>10mm	182	51.4	33	36.3	214	48.1
Timing of surgery						
Primary (before other tx)	246	69.5	84	92.3	330	74.2
Secondary	108	30.5	7	7.7	115	25.8
Radiation therapy						
Received	203	57.3	66	72.5	265	59.6
Defaulted	32	13.6	16	17.6	52	11.7
Not prescribed	119	33.6	7^∏^	7.7	128	28.8

^a^ The percentages in the Totals row are row percentages. The other percentages are column percentages.

*17 patients were missing data on tumor grade.

**3 patients were missing data on histology.

***24 patients were missing data on molecular subtype.

^†^14 patients were missing data on ER and 16 patients on PR expression.

^‡^72 patients were missing data on HIV status.

^↨^86 patients were missing data on lymphovascular invasion.

^Δ^ 42 patients were missing data on closest invasive margins.

^∏^ Had completion mastectomy within 6 weeks

Nearly 40% of surgical patients were younger than 50 years of age, and more than 90% were of black African descent. Women younger than 40 years of age were more likely to receive BCS than those over 60 years. Of 373 surgical patients with known HIV status, 59 (15.5%) were HIV-positive.

Seventeen of 32 patients with stage I disease (53.1%), 195 of 262 with stage II disease (74.4%), and 142 of 151with stage III disease (94.0%) had a TM (p<0.001). Patients with lymphovascular invasion (p = 0.02), a feature of aggressive disease, and those with the HER2-enriched intrinsic subtype (p = 0.02) appear to have been more likely to receive TM than patients without those features.

**Fig 1:**
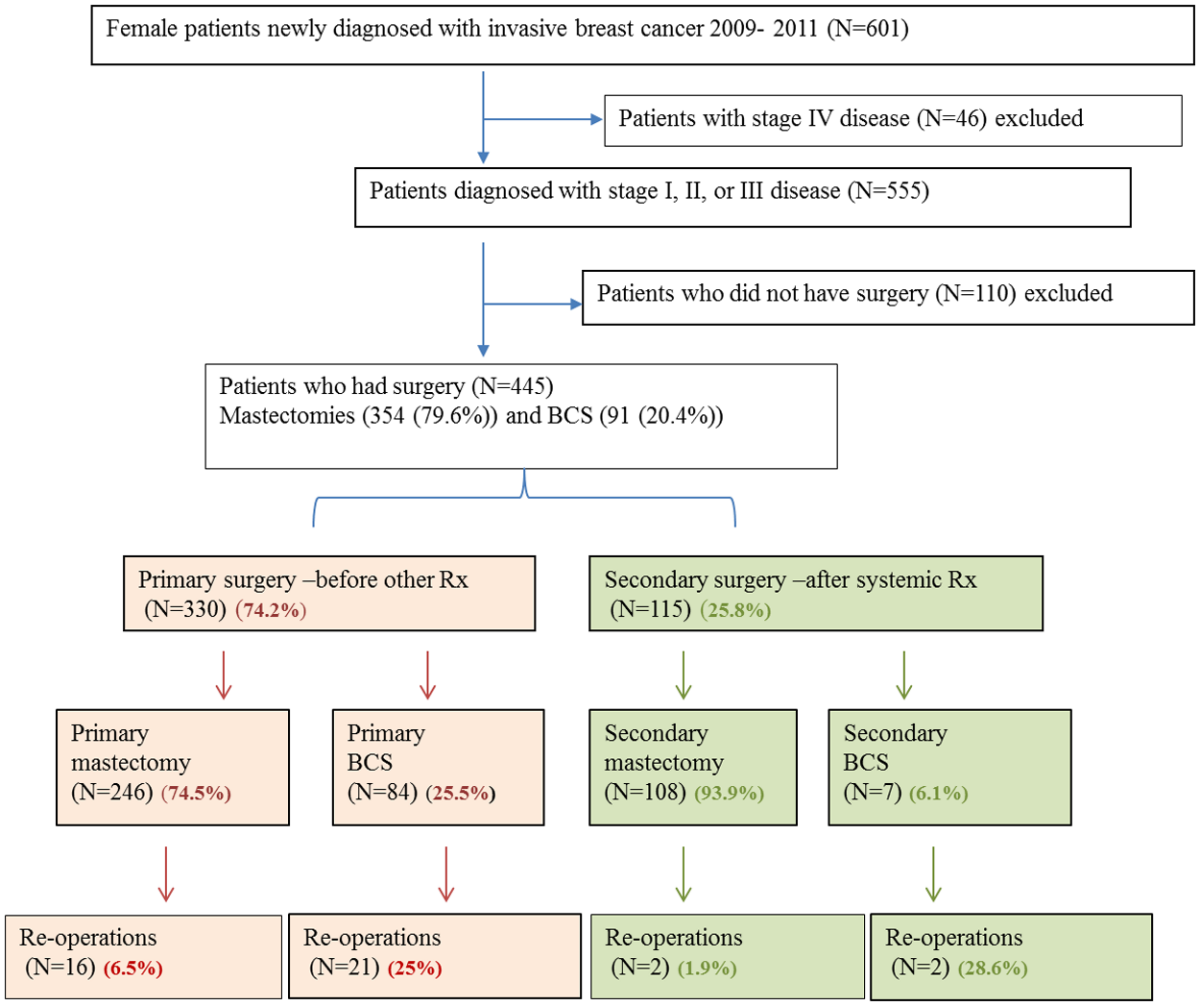
Patients excluded from and included in the analysis of women with breast cancer who underwent surgery at CHBAH, Soweto, South Africa, 2009–2011.

Of the 445 surgically treated patients, 41 (9.2%) underwent re-operation within 6 weeks after the first surgery; 38 reoperations (92.6%) were due to close or involved margins. Other reasons were poor cosmesis, positive sentinel lymph node, or inadequate axillary lymph node dissection (some cases involved more than one reason). Of the 41 patients, 23 (56.1%) had had BCS and 18 (43.9%) TM (p<0.001) (Fig 1).

Of the 354 patients who underwent TM, 235 (66.4%) were referred for RT, of whom 203 (86.4%) received it. In the BCS group of 91patients, 7 had a completion mastectomy (mastectomy performed after BCS because of a problem, usually close or involved margins) within 6 weeks and were not referred for RT. The remaining 84 were referred for RT of whom 66 (78.6%) received it. The referred patients who did not receive RT (32 post TM and 18 post BCS) either defaulted, had change of treatment plan, progressed, died or had untraceable records.

On univariate analysis at p<0.1, age (p = 0.029), stage at diagnosis (p <0.001) and lymphovascular invasion (p = 0.0224) were associated with type of surgical procedure. HIV, race, tumor grade ductal vs. lobular histology and intrinsic subtype (luminal A, luminal B. HER2 positive and triple negative) were not associated.

In a multivariable logistic regression model, older age group (50–59 (p = 0.029) and ≥70 (p<0.001) vs. age group < 40), stage at diagnosis (stage II (p = 0.002) and stage III (p<0.001) vs. stage 1), HIV (HIV positive (p = 0.13) vs. HIV negative) and intrinsic subtype (HER2-enriched subtype (p = 0.018) vs. triple negative) were independently associated with TM (Table 2).

**Table 2:** Odds ratios (OR) and 95% confidence intervals (CI) for the association of demographic and clinical factors with receipt of TM vs BCS.

	OR	95% CI	p-value
Age	1.1	1.0–1.1	<0.001
HIV status			
Negative	1.0	Referent	
Positive	3.7	1.3–7.9	0.005
Unknown	1.6	0.8–3.3	0.22
Stage at diagnosis			
I	1.0	Referent	
II	3.5	1.6–8.7	0.003
III	26.7	9.0–78.6	<0.001
Molecular subtypes			
Triple negative	1.0	Referent	
Luminal A	1.3	0.5–3.4	0.65
Luminal B	1.7	0.9–3.2	0.11
HER2-enriched	3.5	1.2–10.0	0.02

Stage at diagnosis was the strongest predictor of TM.

HIV status was not associated with stage but with age at diagnosis (data not shown).

## Discussion

In this cohort of patients who underwent surgery for breast cancer at a public hospital in Soweto, South Africa, we found that only ~20% received BCS. Factors associated with TM were later stage breast cancer, older age, and HIV infection.

A recent study showed that, in the United States, among women with early stage (I or II) breast cancer, 58% underwent BCS, 36% TM, 3% radiation or chemotherapy without surgery, and about 2% no treatment.[22] However, among our patients in South Africa, 72% of women with early stage breast cancer underwent TM and only 28% received BCS. In the United States, 58% of women with stage III breast cancer undergo TM because their large tumors make it difficult to achieve an acceptable oncologic or cosmetic outcome; only 14% of women diagnosed with stage III disease receive BCS. Among our South African patients with stage III disease, 94% received TM and 6% BCS. In higher-income countries, many patients prefer BCS, and most patients with early disease or a good response to neoadjuvant chemotherapy are considered good BCS candidates.

Doctors in our specialist CHBAH breast unit also have a preference for BCS. However, we need to acknowledge the patient’s wishes and engage each patient individually in the surgical decision making process. Reasons to opt for TM include absolute and relative contra-indications (e.g., inflammatory carcinomas and multi-centric tumors, late stage, and large lesions), major socio-economic obstacles for patients (e.g., out-of-pocket transport costs to the RT center at CMJAH for the 3 or 6 week course of regular or hypo-fractionated treatment with 5 visits per week, or time conflict with job or family demands), patient preferences, and often the desire of both doctors and patients to keep treatment plans as simple as possible. In our resource-constrained health care system, numerous long queues for public transport, patient records, consultations, and medication, and delayed lead times for treatment appointments are major obstacles to patients’ adherence to treatment.

In our model, taking age, stage, and intrinsic subtype into account, patients with HIV were more likely to undergo TM than those who were HIV-negative. Even if surgeons did not consciously influence that choice, patients with HIV might have preferred TM so as to avoid having to travel to CMJAH for RT when they were already receiving ART in another clinic or department.

We had no data about income, but we speculate that patients with HIV may have been poorer than other patients. Poverty is known to be a strong predictor of HIV status and HIV-related medical complications. Poorer patients may choose TM because they cannot manage the additional hospital visits or afford the transportation required to receive adjuvant RT following BCS. [23] Lastly, patients with HIV are more likely to have other medical complications, such as concurrent infections, and to be taking medications, such as ART, which may make additional treatment more burdensome. HIV-infected patients may also have been more likely to be responsible for the care of others, including young children or other family members with HIV and/or tuberculosis.

In recent years, TM has regained some popularity in the United States, in part due to the growing acceptance of prophylactic contralateral TM, increased access to breast reconstruction surgery, improved cosmetic results of such surgery, and increasing use of breast MRI and genetic testing[24].

In the United States, patient-related factors associated with the use of TM include older age, fear of recurrence, perceived survival benefits, previous contralateral TM, medical comorbidities, insurance coverage, and cultural preferences.[25] In a large SEER-based analysis, factors correlated with the decision to undergo BCS as opposed to TM included younger patient age, higher socioeconomic status, favorable tumor characteristics, black race, and urban location. In another study at a single center in Kentucky, race was not independently associated with the choice of BCS versus TM.[26]

In select circumstances where risk for local recurrence is considered high, TM may be indicated for patients with localized disease because local recurrence is associated with a higher risk of death from breast cancer. In the absence of guidelines specifying a risk threshold, a review article concluded that BCS is permissible if the patient’s estimated risk of local recurrence is less than 1% per year, or less than 10% at 10 years. Important risk factors for breast cancer recurrence are: positive margins, young age, decision not to pursue radiotherapy, and intrinsic subtype. The review article also recommended exercising extreme caution when choosing BCS for patients who are younger than 35 years of age; have extensive DCIS (particularly if younger than 40 years of age); have incompletely resected invasive or *in situ* cancer; or are ineligible for radiation therapy (RT). Features of the cancer thought to indicate high risk, but not considered contraindications to BCS, were: multifocality, multicentricity, retroareolar location, vascular invasion, and lobular histology.[27] Generally, BCS was not recommended if it could not achieve a good cosmetic outcome or, more importantly, complete tumor excision.

A clear disadvantage of BCS is the higher risk of requiring a second operation, mainly because of positive margins or those deemed to be unsafe. Twenty-five percent of our BCS patients with early stage breast cancer underwent a second operation within 6 weeks, most often a completion TM. This high re-operation and TM rate was largely due to decision making in our multidisciplinary meetings, where we had a preference for widely clear margins above 5mm. Nearly 19% of specimens from our patients who had BCS had 1–5 mm margins that were in many cases the indication for re-operation. Had we followed current guidelines[28, 29], our BCS re-operation rate would have been below 15%. Furthermore, close margins were often unexpected areas of associated DCIS that could not be identified by pre-operative imaging; mammography is known to underestimate the presence and the extent of DCIS.

Our data show that at least 80% of our referred patients received RT. Although they had to travel ~18 kilometers from their place of residence for that treatment, they had better access to RT than patients in most parts of sub-Saharan Africa, where very limited facilities exist.[30] Nevertheless, some patients who are eligible for BCS from a surgical perspective may opt for TM in order to obviate the need for adjuvant RT.

Studies from other countries in sub-Saharn Africa report that many patients, regardless of age and educational background, refuse TM which may be the only surgical option even though they understand that they may have cancer and that their condition may otherwise be lethal. In one study of Nigerian breast cancer patients, 44.7% of patients delayed TM because of fear.[31] Patients feared disfigurement, effect on relationships with a partner, and surgical complications leading to extended duration or escalation of care.[32]

Interestingly, among women in Switzerland who were diagnosed with operable breast cancers but declined surgery, common reasons included depression, desire to pursue alternative therapies, medical comorbidities, fear, age, and inadequate insurance.[33]

Our study is one of the first to describe HIV status in relation to surgery for breast cancer in South Africa. We have studied a population in South Africa with a high prevalence of HIV infection and with access to ART and standard breast cancer treatments. Limitations include the relatively small sample size, the lack of data on socioeconomic status, and the lack of follow-up for long-term outcomes, such as survival. In addition, our analysis drew on what was originally an administrative database; as such it may have been affected by bias or confounding. We have initiated a prospective multi-center collaborative South African outcome study to address these issues. However, meanwhile our efforts have taught us that an oncoplastic approach in breast surgery is feasible even in a resource constrained setting, and that zero tolerance of long waits for surgery reduces the surgical workload and minimizes loss to treatment.

## Conclusions

Patients with breast cancer in our cohort from South Africa, even those with stage I disease, were more likely than patients in more affluent countries to have a TM than BCS. Stage at diagnosis was the strongest determinant of type of surgery, but HIV status also appeared to be a determinant of surgical procedure (probably by affecting patients rather than doctors’ decision making). We found also a possible association with HER2 enriched status and presence of lymphovascular invasion, both indicators of aggressive disease. The reasons for these associations need to be better understood, as do the reasons for the choice of TM when BCS is a valid option. Although our relatively low BCS numbers and high re-operation rates reflect the challenges of offering BCS in a poorly resourced environment, we believe it is worth the effort. One could argue that we are adding complexity to an already strained health system, but BCS has the potential to change perceptions and alleviate the fears of surgical mutilation associated with breast cancer. BCS may be a potential catalyst for women to come forward with earlier disease.
